# A Small Subunit of Geranylgeranyl Diphosphate Synthase Functions as an Active Regulator of Carotenoid Synthesis in *Nicotiana tabacum*

**DOI:** 10.3390/ijms24020992

**Published:** 2023-01-04

**Authors:** Chen Dong, Mei Zhang, Shanshan Song, Fang Wei, Lili Qin, Puqing Fan, Yongchun Shi, Xiaoran Wang, Ran Wang

**Affiliations:** 1 College of Life Sciences, Henan Agricultural University, Zhengzhou 450002, China; 2 College of Biological Engineering, Henan University of Technology, Zhengzhou 450001, China; 3 School of Agricultural Sciences, Zhengzhou University, Zhengzhou 450001, China; 4 College of Tobacco Science, Henan Agricultural University, Zhengzhou 450002, China

**Keywords:** carotenoid, geranylgeranyl diphosphate synthase (GGPPS), methylerythritol phosphate pathway (MEP), phytoene synthase, small subunit (SSU)

## Abstract

As one of the most imperative antioxidants in higher plants, carotenoids serve as accessory pigments to harvest light for photosynthesis and photoprotectors for plants to adapt to high light stress. Here, we report a small subunit (SSU) of geranylgeranyl diphosphate synthase (GGPPS) in *Nicotiana tabacum*, NtSSU II, which takes part in the regulation carotenoid biosynthesis by forming multiple enzymatic components with NtGGPPS1 and downstream phytoene synthase (NtPSY1). NtSSU II transcript is widely distributed in various tissues and stimulated by low light and high light treatments. The confocal image revealed that NtSSU II was localized in the chloroplast. Bimolecular fluorescence complementation (BiFC) indicated that NtSSU II and NtGGPPS1 formed heterodimers, which were able to interact with phytoene synthase (NtPSY1) to channel GGPP into the carotenoid production. CRISPR/Cas9-induced *ntssu II* mutant exhibited decreased leaf area and biomass, along with a decline in carotenoid and chlorophyll accumulation. Moreover, the genes involved in carotenoid biosynthesis were also downregulated in transgenic plants of *ntssu II* mutant. Taken together, the newly identified NtSSU II could form multiple enzymatic components with NtGGPPS1 and NtPSY1 to regulate carotenoid biosynthesis in *N. tabacum*, in addition to the co-expression of genes in carotenoids biosynthetic pathways.

## 1. Introduction

Terpenoids, including monoterpene, sesquiterpene, diterpene, triterpene, and tetraterpene, are a kind of secondary metabolites with the most quantitative and structural variations in plants [1,2,3,4]. Terpenoids are widely involved in plant growth and development, photosynthesis, signal transduction, environmental adaptation, and stress resistance [5,6,7,8]. Among the main groups of terpenoids, carotenoids function as essential components of the photosynthetic system and light-absorbing apparatus in plastids [9,10,11,12]. In addition, carotenoid also provides precursors for the biosynthesis of phytohormones such as abscisic acid (ABA) and strigolactones (SLs) [13,14]. Terpenoids derived from isopentenyl pyrophosphate (IPP) and its allyl isomer dimethylallyl pyrophosphate (DMAPP) are subsequently used to generate prenyl diphosphates of increasing size, including geranyl diphosphate (GPP), farnesyl diphosphate (FPP), and geranylgeranyl diphosphate (GGPP). IPP and DMAPP can be synthesized in two biosynthetic pathways: the mevalonic acid (MVA) pathway in the cytoplasm and the 2-C-methyl-D-erythritol 4-phosphate (MEP) pathway in the plastid [1,10,15,16,17]. GGPP is the common precursor of various terpenoids, including diterpenes, gibberellins (GAs), α-tocotrienol, chlorophyll, and carotenoid [18,19,20,21]. GGPP synthase (GGPPS) is involved in allocating GGPP to downstream enzymes for the synthesis of various terpenoids [3,18,19], which consists of two aspartate-rich domains, first aspartate rich motif (DDxxxxD, FARM) and second aspartate rich motif (DDxxD, SARM) [22,23]. GGPPS can be classified into five subgroups, Sub I, Sub II, Sub III, Sub IV, and Sub V [24]. Among five subgroups, Sub I, Sub II, Sub III, and Sub V belong to the large subunit (LSU) of GGPPS, which form homodimers to synthesize GGPP or GPP, while Sub IV is named as small subunit (SSU) of GGPPS including SSU I and SSU II. Different from LSU, SSU I lacks the FARM and SARM domains and contains two CxxxC domains [25,26], while the SSU II proteins contain the FARM and two CxxxC domains [27]. The SSU without enzymatic activity serves to modify catalytic fidelity or to promote catalytic activity by binding LSU in plants [18,27,28].

After the combination of SSU I and the corresponding LSU, the catalysis product of the latter was modulated from the synthesis of GGPP to GPP, thereby allowing the GGPP channeling to monoterpene biosynthesis. For example, over-expression of *MpSSU I* in *Mentha piperita* significantly increased the content of GPP and monoterpenes in tobacco plants, with a decline of β-carotene [29]. Additionally, SSU I has been successively isolated and identified in *Antirrhinum majus* and *Clarkia breweri*, and their functions are similar to *MpSSU I* [26].

The HlLSU homodimers in *Humulus lupulus* can catalyze the production of GPP (26.9%), FPP (4.9%), and GGPP (68.2%), and the HlSSU I has no catalytic activity. When the heterodimers of HlLSU-HlSSU II are formed, GPP (59.5%) and GGPP (40.5%) can be synthesized using IPP and DMAPP as substrates. Therefore, the activity of HlLSU-HlSSU I heterodimers is significantly higher than HlLSU homodimers [27]. Moreover, AtSSU II in *Arabidopsis thaliana* can form heterodimers with GGPPS-LSU proteins (AtGGPPS11, AtGGPPS2, and HlLSU) to improve GPP production, which indicates that AtSSU II functions similarly compared to HlSSU I. Particularly, AtGGPPS2 homodimers only produce GGPP, yet it obtains the ability to synthesize trace GPP after forming heterodimers with AtSSU II [27]. Different from MpSSU I and AtSSU II, the GGPPS recruiting protein (GRP) in rice, which belongs to the SSU II subfamily, is involved in allocating GGPP to chlorophyll synthetic pathway [18]. Compared with OsGGPPS1 (LSU) homodimers, OsGRP-OsGGPPS1 heterodimers produced more GGPP for chlorophyll synthesis [18]. The OsGRP-OsGGPPS1 heterodimers can recruit and form protein complexes with geranylgeranyl reductase (OsGGR), light harvesting like protein 3 (OsLIL3), protochlorophyllide oxidoreductase (OsPORB), and chlorophyll synthase (OsCHLG), which are crucial enzymes in the early phase of chlorophyll synthetic pathway. However, both OsGGPPS1 and OsGRP have no interaction with phytoene synthase (OsPSY), the first enzyme of the carotenoid synthetic pathway, converting GGPP to phytoene [18]. Only CaSSU II in *Capsicum annuum* can bind CaPSY and CaGGPPS1 (LSU) and plays key roles in ripening fruit development and capsanthin production [30].

The biological role of SSU II is still needed to be unveiled in higher plants, especially in the polyploid plants containing multiple members of SSUs, GGPPS, and PSYs. *Nicotiana tabacum* has been classically used as an efficient system for studying carotenoid biosynthetic pathways in higher plants [19,31,32,33,34]. Our previous study indicated that *N. tabacum* comprises multiple members of GGPPS [19] and PSY [35]. Carotenogenic genes have been widely expressed in tobacco to improve its carotenoid content and photosynthetic efficiency [19]. 

To address this question, tobacco was used as a polyploid plant model system. We demonstrated that both SSU I and SSU II exist in *N*. *tabacum*, which are classified as NtSSU I-1, NtSSU I-2, and NtSSU II. Bimolecular fluorescence complementation (BiFC) indicated that NtSSU II and NtGGPPS1 are able to form heterodimers, which interact with NtPSY1 to channel GGPP into the production of carotenoids. Exploring the regulation of GGPPS activity by NtSSU II has great theoretical significance in improving the carotenoid content and quality of crops, providing a theoretical foundation for the genetic regulation of photosynthesis efficiency.

## 2. Results

### 2.1. Characteristic and Phylogenetic Analysis of NtSSU Family in N. tabacum

**Figure 1:**
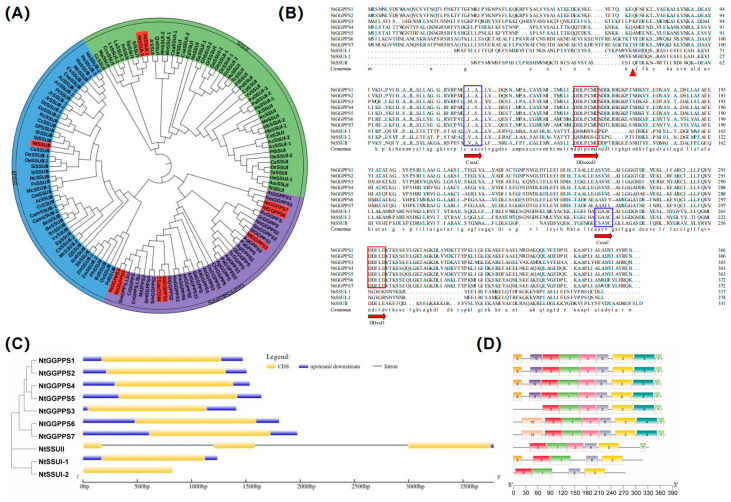
Sequence alignment and phylogenetic analysis of GGPPS family. (**A**) Phylogenetic analysis of GGPPS family. GGPPS family was classified as subgroups of SSU I, SSU II, GGPPS, and GPPS in high plants. (**B**) Sequence analysis of NtGGPPSs and NtSSU. FARM and SARM motifs considered as the binding and catalysis sites were in red boxes. The two conserved domains CxxxC were in blue boxes, which was essential for the interaction of the SSU II protein with other proteins. The putative transit peptides truncated were in red triangle. (**C**,**D**) The exon-intron structure and conserved motifs in NtGGPPSs and NtSSU. The protein sequences were from NCBI database. *NtGGPPS1*, NP001312106.1, *NtGGPPS2*, XP016435651.1, *NtGGPPS3*, NP001313025.1, *NtGGPPS4*, XP009784771.1, *NtGGPPS5*, NP001312601.1, *NtGGPPS6*, XP009784772.1, *NtGGPPS7*, XP009627577.1, *NnGGPPS1*, XP010254951.1, *NnGGPPS2*, XP010264689, *AgGPPS1*, AAN01133.1, *AgGPPS2*, AAN01134.1, *AgGPPS3*, AAN01135.1, *PaGPPS*, ACA21458.2, *MiGPPS1*, AFJ52721.1, *AtGGPPS2*, NP_195399.1, *MiGPPS2*, AFJ52722, *CrGGPPS*, AGL91645.1, *VvGPPS*, AAR08151.1, SlGGPPS1, NP001234087, *SlGGPPS2*, ABB82555.1, *SaGGPPS*, Q43133.1, *OsGPPS*, KAB8080775.1, *OsGGPPS1*, KAB8080775.1, *SlGPPS*, ABB88703.1, *QrGPPS*, CAC20852.1, *SmGPPS*, AEZ55677.1, CrGPPS.LSU, AGL91645.1, SmGPPS.LSU, AEZ55681.1, AtGGPPS1, NP_175376.1, SmGGPPS3, AEZ55683.1, *AtGGPPS4*, NP_179960.1, *AtGGPPS3*, NP_179454.1, *SmGGPPS2*, AEZ55682.1, *JcGGPPS*, ADD82422.1, *RcGGPPS*, XP_002531191.1, *GbGGPPS*, AAQ72786.1, *TcGGPPS*, AAD16018.1, *AgGGPPS*, AAL17614.2, *PaGGPPS5*, ACA21461.1, *ChrGGPPS*, EDO96545.1, *PaGGPPS6*, ACA21462.1, *SmGGPPS4*, ACR19637.1, *SmGGPPS1*, ACJ66778.1, *NtSSUI-1*, XP016455366.1, *NtSSUI-2*, XP_016465609.1, *NtSSUII*, NP001312125.1, *CaSSUII*, XP027061108, *SlSSUI*, XP010324311, *SpSSUI*, XP015081852, *SPSSUII*, NP001310395, *CeSSUI*, XP027170690, *TcSSUI*, XP007020796, *QsSSUI*, XP023888195, *OeSSUI-1*, XP022867418, *OeSSUI-2*, XP022880479, *OeSSUII-1*, XP022898674, *OeSSUII-2*, XP022876147, *AcSSUI-1*, PSR95599, *AcSSUI-2*, PSR85329, *AcSSUII-1*, PSS29542, *AcSSUII-2*, PSS21781, *TaSSUI*, AUZ98418, *DsSSUI*, XP017249855, *DzSSUI-1*, XP022734589, *DzSSUI-2*, XP022753925, *DzSSUII-1*, XP022725260, *DzSSUII-2*, XP022763000, *PlSSUI*, AKJ26303, *SiSSUI*, XP011095406, *SiSSUII*, XP011092951, *JcSSUI*, XP012070840, *HuSSUI*, XP021287931, *MnSSUI-1*, EXB38722, *MnSSUI-2*, -XP010089993, *MnSSUII*, XP010102651, *PeSSUI*, XP011007747, *PtSSUII*, XP002312936, *PtSSUI*, XP024442001, *LsSSUI-1*, XP023764268, *LsSSUI-2*, XP023764267, *HbSSUI-1*, XP021692305, *HbSSUI-2*, BAF98300.1, *MeSSUI*, XP021594137, *CsaSSUI-1*, XP030506961, *CsaSSUI-2*, XP030510759, *CsaSSUII*, XP030483625, *GhSSUI-1*, XP016701785, *GhSSUI-2*, XP016732469, *GaSSUI-1*, XP017604285, *GaSSUI-2*, XP017640996, *GrSSSUI*, XP012460748, *RcSSUI*, XP002532570, *RcSSUII*, XP002529802, *JrSSUI*, XP018811644, *JrSSUII-2*, XP018841527, *JrSSUII-1*, XP018853159, *VvSSUI*, XP002278023, *VvSSUII*, XP003631563, *CpSSUI*, XP021905820, *CcsSSUII*, XP024960715, *PaSSUI-3*, XP028756705, *PaSSUI-2*, XP028806557, *PaSSUI-1*, XP028756707, *EgSSUI*, XP010060334, *EgSSUII*, XP010066312, *CcSSUI*, XP006452381, *CcSSUII*, XP_006422513, *InSSUI*, XP019196842, *InSSUII*, XP019150720, *AtSSUII*, AT4g38460, *OsSSUII*, XP015626863, *PhSSUII*, XP025804311, *SbSSUII*, XP002452775, *ZmSSUII-1*, XP008678927, *ZmSSUII-2*, PWZ23732, *BdSSUII*, XP003570062, *PdSSUII*, XP008813057, *ZjSSUII*, XP015893240, *BnSSUII*, XP013658388, *CamSSUII-1*, XP010436939, *CamSSUII-2*, XP010431779, *CrSSUII*, XP023635209, *BoSSUII*, XP013609863, *RsSSUII*, XP018449141, *HaSSUII-1*, XP021988672, *HaSSUII-2*, XP021976342, *HaSSUII-3*, XP021976340, *RcSSUII*, XP024165175, *McSSUII-1*, XP022136229, *McSSUII-2*, XP022140742, *FvSSUII*, XP004290755, *StSSUII*, XP006341179, *CmSSUII*, XP022928137, *HlSSUI*, ACQ90681.1, *MpSSUI*, AAF08792.1, *AmSSUI*, AAS82859.1, *SlSSU II*, NP001353635, *NnSSUI*, XP010276119, *NnSSUII*, XP010266070.1.

Screening the tobacco genome revealed ten candidate genes with sequence similarities to the GGPPS genes of *A. thaliana* or rice (Table S1). Seven genes including *NtGGPPS1*, *NtGGPPS2*, *NtGGPPS3*, *NtGGPPS4*, *NtGGPPS5*, *NtGGPPS6,* and *NtGGPPS7* encode large subunit (LSU) of NtGGPPS, and two genes as *NtSSU I-1* and *NtSSU I-2* belong to SSU I, one gene as *NtSSU II* is classified as SSU II. Phylogenetic analysis indicated that NtSSU II was closely clustered with SSU II in *C. annuum* and *Solanum lycopersicum*, which all belong to Solanaceae. Monocotyledons such as *Oryza sativa*, *Zea mays*, and *Sorghum bicolor* normally consist of SSU II. Dicotyledons, including *C. annuum*, *S. lycopersicum*, *Juglans regia*, *Ricinus communis*, *Morus notabilis*, and *Durio zibethinus* contain two types of SSU (SSU II and SSU I) (Figure 1A). Sequence alignment indicated that the first and second aspartate-rich (FARM and SARM) motifs, considered the binding and catalysis sites in prenyltransferases, were detected in NtSSU II. Moreover, almost all SSU II comprise two conserved domains (CxxxC), which is essential for the interaction of the SSU II protein with other proteins (Figure 1B). The exon-intron architecture analyzed by GSDS 2.0 software indicates that the *NtGGPPS* and *NtSSU I* have no introns. NtSSU II comprises two introns and three exons (Figure 1C).

### 2.2. Expression Pattern of NtSSUs

**Figure 2:**
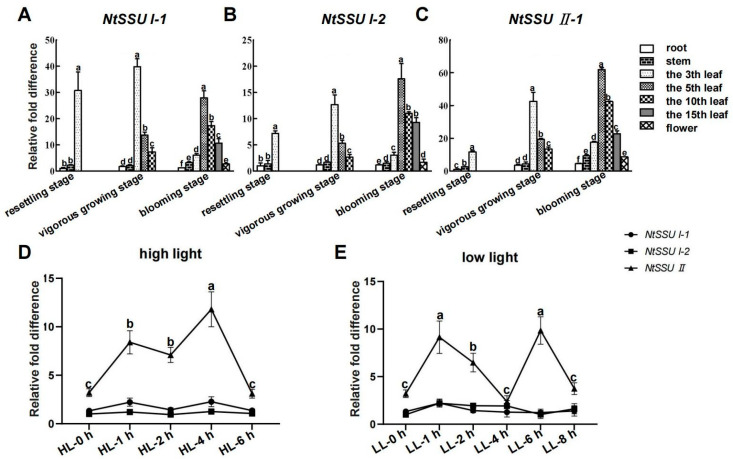
The expression profiles of *NtSSU II*, *NtSSU I-1*, and *NtSSU I-2* in various growth stages and in response to light treatments. (**A**–**C**) Different tissues including root stem, the 3rd leaf, the 5th leaf, the 10th leaf, the 15th leaf, and flower were harvested at resettling stage, vigorous growing stage, and blooming stage, respectively. (**D**) Four weeks-tobacco plants were selected for high light (1000 μmol m^−2^ s^−1^) treatment. The leaves were collected after 1 h high light treatment (HL-1 h), HL-2 h, HL-4 h, and HL-6 h, respectively, using no treatment tobacco as control (HL-0 h). (**E**) Four weeks-tobacco plants were selected for low light (100 μmol m^−2^ s^−1^) treatment. The leaves were collected after 1 h low light treatment (LL-1 h), LL-2 h, LL-4 h, LL-6 h, and LL-8 h, respectively, using no treatment tobacco as control (LL-0 h). Relative expression was evaluated by qPCR using 26s rRNA as a reference gene. Three biological replicates were applied for each experiment. Data are presented as means ± SEM (n = 3 biological replicates). Different letters represent significant differences at *p* < 0.05; one-way ANOVA.

The expression pattern of the three *NtSSUs* is similar at the three developmental stages. At resettling stage, the third leaf exhibited a higher mRNA level of *NtSSU I-1*, *NtSSU I-2*, and *NtSSU II* than root and stem (Figure 2A–C). The highest expression of *NtSSU I-1*, *NtSSU I-2*, and *NtSSU II* was also detected in the third leaf at vigorous growing stage, and the expression of three transcripts of *NtSSUs* in the fifth leaf was more than the tenth leaf. Unlike their expression profiles described above, the three transcripts of the fifth leaf exhibited the highest expression pattern at blooming stage, and a higher mRNA level in the tenth leaf was detected than in the fifteenth leaf. While the transcripts of *NtSSU I-1*, *NtSSU I-2*, and *NtSSU II* in the fifth leaf, tenth leaf, and fifteenth leaf were higher than in the third leaf (Figure 2A–C). Moreover, qPCR indicated that high light treatment augmented expression of NtSSU II after 1 h high light treatment (HL-1 h), with the highest mRNA level after HL-4 h. Then *NtSSU II* transcript was decreased after HL-6 h. However, no change of *NtSSU I-1* and *NtSSU I-2* transcripts was detected caused by high light treatment (Figure 2D). Compared to no change of *NtSSU I-1* and *NtSSU I-2* transcripts during low light treatment, the expression of *NtSSU II* was increased after 1 h low light treatment (LL-1 h) and then downregulated after LL-2 h and LL-4 h (Figure 2E). Finally, the mRNA level increased after LL-6 h and decreased after LL-8 h (Figure 2E). 

### 2.3. Subcellular Location of NtSSU

**Figure 3:**
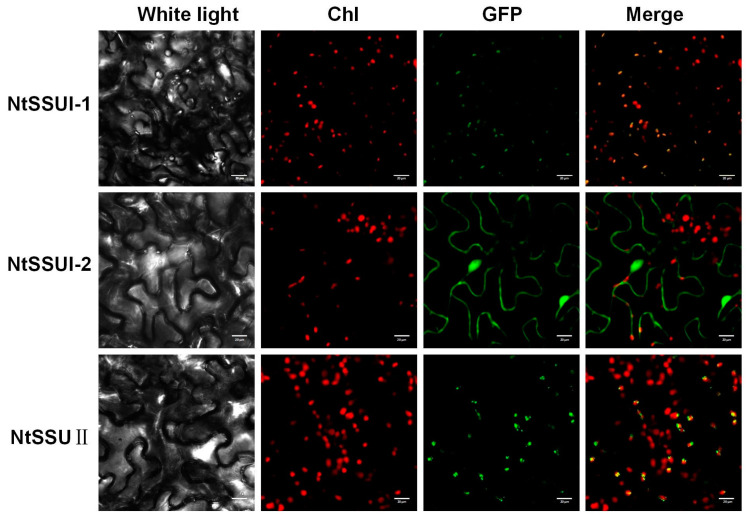
Subcellular localization of NtSSUs. Subcellular location of NtSSU II, NtSSU I-1, and NtSSU I-2 proteins fused to GFP in tobacco. Chl, chlorophyll autofluorescence.

To investigate their subcellular locations, the ORFs of *NtSSU II*, *NtSSU I-1*, and *NtSSU I-2* were fused to the N terminus of GFP and transiently expressed in *Nicotiana benthamiana* leaves (Table S2). GFP signals indicated that the NtSSU II and NtSSU I-1 were mainly distributed in the chloroplast as well as the NtSSU I-2 was mainly distributed in the cytoplasm (Figure 3).

### 2.4. Bacterial Pigment Complementation Assay of NtSSU II

**Figure 4:**
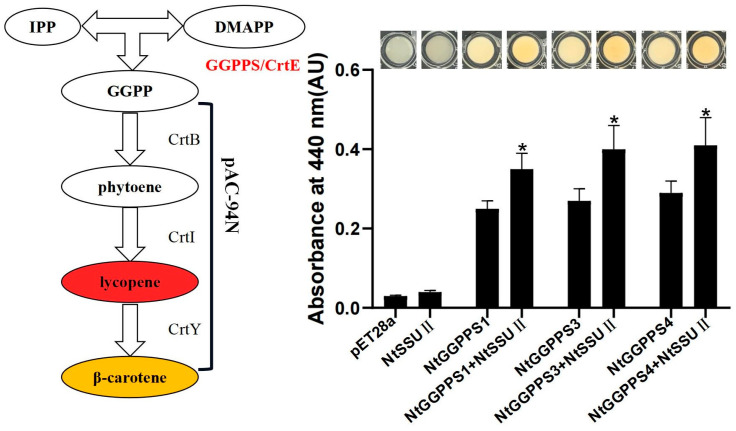
Complementation assays of NtSSU II. To determine NtSSU II activity, the recombinant vectors of pET28a-NtSSU II were co-transformed with pET32b-NtGGPPSs and pAC-94N into *E. coli* BL21(DE3) cells, using empty pET28a as negative control. The absorbance at 440 nm was used to estimate the quantity of β-carotene. Asterisks represent statistically significant differences between pET32b-NtGGPPSs and pET32b-NtGGPPSs co-transformed with pET28a-NtSSU II determined by Student’s t-test (*, *p* < 0.05).

Our previous study indicated that NtGGPPS1, NtGGPPS3, and NtGGPPS4 could synthesize GGPP to produce β-carotene among seven NtGGPPSs [19]. The activity of NtSSU II was also examined by a bacterial pigment complementation assay. The pAC-94N vector encoding a gene cluster for β-carotene biosynthesis (Figure 4) were co-transformed with recombinant vector pET28a-NtSSU II in the *Escherichia coli* BL21 (DE3) strain. The GGPP generated by functional GGPP induced to change the bacteria color from white to yellow. In this experiment, *E. coli* only harboring NtSSU II and pAC-94N was not able to produce β-carotene. The yellower color was detected in *E. coli* when co-expression of NtSSU II and NtGGPPS1, NtGGPPS3, or NtGGPPS4, exhibiting higher absorbance at 440 nm than only NtGGPPSs transformation (Figure 4). It suggested that NtSSU II enhanced the activity of NtGGPPSs to generate more β-carotene (Figure 4).

### 2.5. NtSSU II Could Interact with NtPSY1 and NtGGPPS1

**Figure 5:**
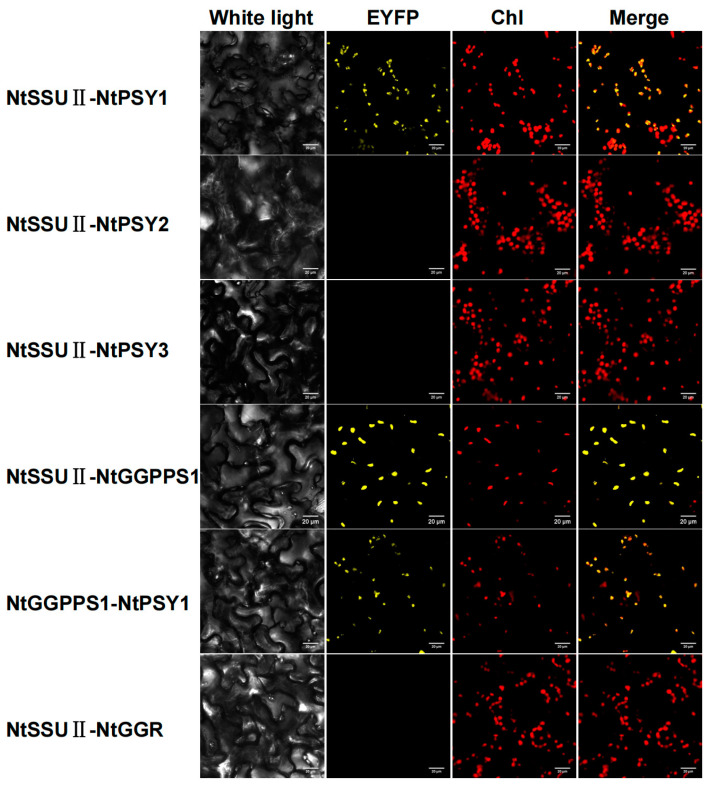
BiFC detection of interactions between NtSSU II proteins and potential partners downstream of the GGPP node. Chl, chlorophyll autofluorescence. Fluorescence of reconstructed EYFP was detected as the result of protein-protein interactions in the chloroplast (Chl).

To unveil the role of NtSSU II, the interactions between NtSSU II and potential enzymes downstream of the GGPP node were examined by BiFC experiment (Figure 5). As the first enzyme downstream of the GGPP in carotenoids biosynthesis, there are three NtPSYs in tobacco named NtPSY1, NtPSY2, and NtPSY3. NtSSU II was highly interacted with NtPSY1 except for NtPSY2 and NtPSY3 in BiFC experiments. Moreover, NtSSU II was also able to interact with NtGGPPS1. There were no protein–protein interactions between NtSSU II and the downstream proteins GGR. NtGGPPS1 can interact with itself in the chloroplast, and there was no evidence for the interaction of other NtGGPPSs with NtPSY1 (Figure 5). 

### 2.6. Phenotypes of ntssu II Mutants and The Regulation Effects of NtSSU II

**Figure 6:**
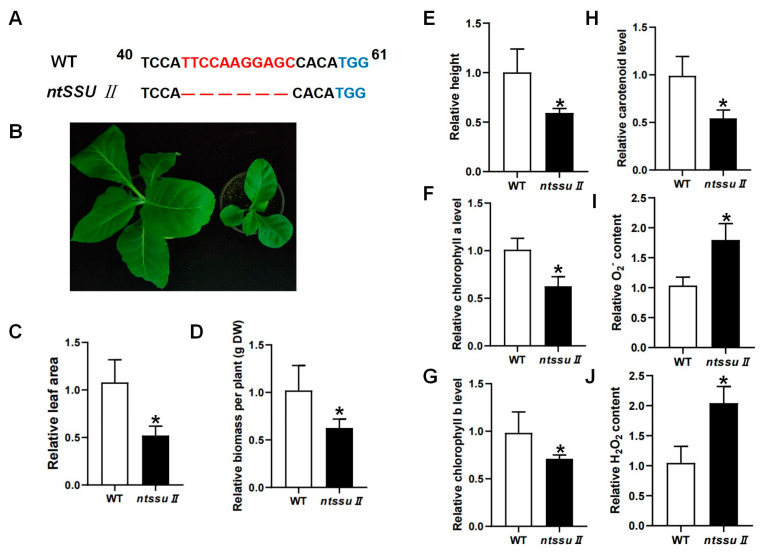
Phenotype of *ntssu II* transgenic tobacco and identification of carotenoids and chlorophyll contents. (**A**) Sequences analysis of *ntssu II* mutant. The TGG indicated in blue was the PAM, and the red fonts represented the nucleotide bases deletion generated by the CRISPR/Cas9 system in *ntssu II* mutants. (**B**) The phenotype of *ntssu II* mutant. (**C**–**E**) Relative leaf area, biomass, and plant height of *ntssu II* mutant. (**F**–**H**) Relative contents of chlorophyll a, chlorophyll b, and carotenoid. (**I**,**J**) The relative contents of O_2_^−^ and H_2_O_2_ contents in *ntssu II* mutant. Asterisks represent statistically significant differences between WT and transgenic plants determined by Student’s t-test (*, *p* < 0.05).

**Figure 7:**
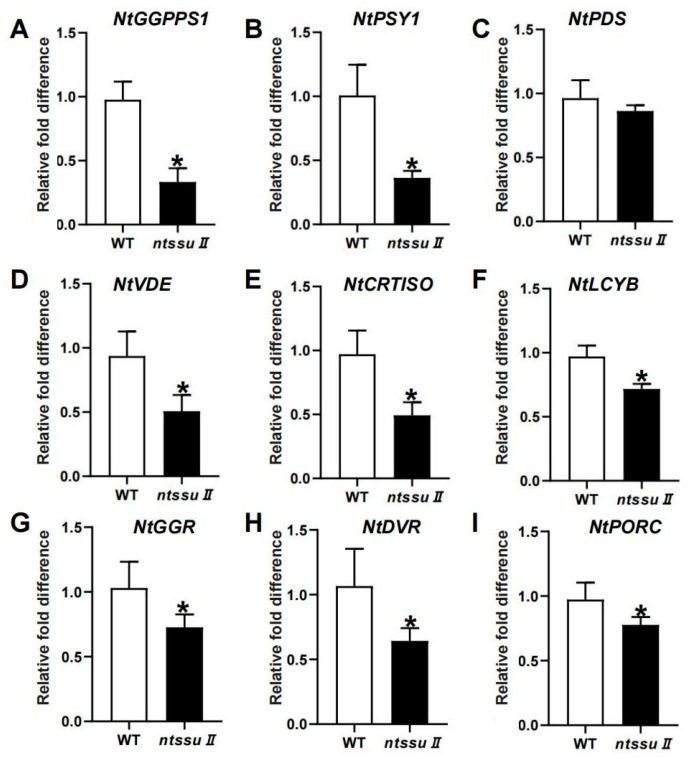
Relative expression of genes involved in carotenoid and chlorophyll biosynthesis. (**A**–**F**) The relative mRNA level of genes related to carotenoid biosynthesis, including *NtGGPPS1*, *NtPSY1*, *NtPDS*, *NtVDE*, *NtCRTISO*, and *NtLCYB* in *ntssu II*, using WT as control. (**G**–**I**) The expression of genes involved in chlorophyll biosynthesis, including *NtGGR*, *NtDVR*, and *NtPORC*. Asterisks represent statistically significant differences between WT and transgenic plants determined by Student’s *t*-test (* *p* < 0.05).

To investigate the functions of the *NtSSU II* genes, CRISPR/Cas9-mediated editing was used to generate *ntssu II* mutants using the MSBSPPCR method [36]. The homozygous mutants were generated successfully (Figure 6A). The target site of NtSSU II was located between 40 and 58 bp from the 5′ to the 3′ end. The *ntssu II* mutants contained a homozygous 11-bp deletion between 44 bp and 54 bp, giving rise to a frame-shift mutation (Figure 6A). Silencing of NtSSU II inhibited the normal growth of tobacco seedlings (Figure 6B), with decreased leaf area, fewer biomass, and lower plant height of *ntssu II* mutants than WT at 40 days after sowing (Figure 6C–E). The chlorophyll contents, including chlorophyll a and chlorophyll b, were also decreased in *ntssu II* mutants (Figure 6F,G). Consistent with the decline in chlorophyll contents, the carotenoid contents were also relatively decreased in *ntssu II* mutants compared with WT (Figure 6H). However, the contents of O_2_^−^ and H_2_O_2_ in transgenic tobacco were increased in *ntssu II* mutants compared to WT (Figure 6I,J). To reveal the regulation effects of NtSSU II and assess the equilibrium associated with *NtSSU II*, we performed qPCR to identify the key genes regulated by NtSSU II (Figure 7). Expression of downstream genes that encode potential enzymes in carotenoid biosynthesis (*NtGGPPS1*, *NtPSY1*, *NtVDE*, *NtCRTISO*, and *NtLCYB*) was decreased in *ntssu II* mutants compared with WT, with no change detected in *NtPDS* (Figure 7A–F). The genes encoding enzymes related to chlorophyll biosynthesis (*NtGGR*, *NtDVR*, and *NtPORC*) were also downregulated in *ntssu II* mutants compared with WT (Figure 7G–I).

## 3. Discussion

Most of the essential plant isoprenoids are derived from GGPP, which is essential for primary and secondary isoprenoid compound synthesis [19,37]. However, the mechanism of allocating GGPP among competing metabolic pathways leading to the formation of these compounds is still unclear. To date, the regulation role of SSU has been only reported in the SSU members in *A. thaliana*, *H. lupulus* [27], rice [18], and *C. annuum* [30]. As a polyploid plant model, our previous result indicated that NtGGPPS1 is involved in the carotenoid biosynthetic pathway of *N. tabacum* among seven NtGGPPSs. Rational design of NtGGPPS1 enhanced carotenoid production and improved photosynthetic efficiency [19]. The biological role of SSU II is still unknown in the polyploid plants *N. tabacum*, which contains multiple members of SSUs, GGPPSs, and PSYs. In this study, we demonstrated that *N. tabacum* consisted of NtSSU I-1, NtSSU I-2, and NtSSU II. The evolutionary relationships and the exon-intron structure of NtSSU were characterized (Figure 1). Among these groups, GGPPS and SSU II exhibit a close evolutionary relationship compared with SSU I-1 and SSU I-2, which might evolve from the same ancestral gene (Figure 1). The conserved motifs of FARM and SARM, considered the binding and catalysis sites in GGPPS, are detected in NtSSU II. Moreover, NtSSU II consists of two conserved domains (CxxxC), which are essential for the interaction of the SSU II protein with other proteins (Figure 1). This evidence clearly indicated that SSU II could participate in the regulation of GGPPS activity.

The *NtSSU II* transcript in leaves was stimulated by high light and low light treatment, with no change of *NtSSU I-1* and *NtSSU I-2* (Figure 2). It suggested that only NtSSU II was in response to light induction among NtSSU I-1, NtSSU I-2, and NtSSU II. NtSSU II roles as the stimulator of carotenoid biosynthesis in response to light treatments. Moreover, the subcellular location suggested that NtSSU I-1, NtSSU I-2, and NtSSU II probably exhibited various roles in tobacco (Figure 3). Bacterial pigment complementation assay illustrated that the inactive NtSSU II enhanced the activity of NtGGPPS1, NtGGPPS3, or NtGGPPS4 (Figure 4).

In addition, our data provided evidence of the existence of multi-enzyme complexes containing NtGGPPS1, NtSSU II, and NtPSY1 in chromoplasts that were specialized in the production and accumulation of high amounts of carotenoids (Figure 5). Three enzymes that channel GGPP to the production of carotenoids were consistent with the finding of CaSSU II [30]. The switch between NtGGPPS1 homodimers and NtGGPPS1-NtSSU II heterodimers was probably part of a regulatory mechanism to balance the production of different types of isoprenoid products. 

The silencing of PSY, PDS, β-LCY, and VDE caused the photobleaching of different plant organs and a significant decline in photosynthetic efficiency and stress resistance [35,38,39,40]. Unlike these genes in the carotenoid pathway, CRISPR/Cas9-mediated NtSSU II silencing hampered the normal growth of transgenic tobacco, with a decline in leaf area, plant height, and biomass (Figure 6). The relative Chl a and Chl b contents were also less in the *ntssu II* mutants than in WT. Carotenoid content exhibited the same trend as leaf growth and chlorophyll contents. NtSSU II mutation attenuated plant carotenoid accumulation, resulting in ROS accumulation compared to WT (Figure 6). Consistent with the role of NtSSU II, silencing of CaSSU II also decreased carotenoid contents in pepper [30].

**Figure 8:**
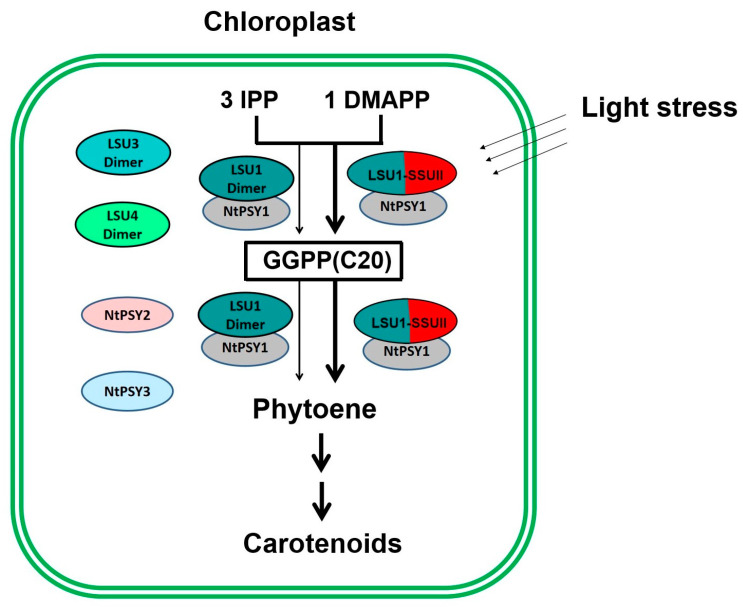
Schematic overview of NtSSU II roles in enhancing carotenoid biosynthesis in Chloroplast. Three members of LSU in tobacco can synthesize GGPP using IPP and DMAPP as substrates. NtSSU II is able to interact with LSU1 (NtGGPPS1) and NtPSY1 that forming the enzyme complex to guide GGPP toward carotenoid biosynthesis. NtSSU II roles as regulator of NtGGPPS1, which exhibited higher enzymatic activity than LSU1 homodimers under light stress.

As the gene regulating NtGGPPS1, NtSSU II is highly co-expressed with genes encoding carotenoid and chlorophyll biosynthesis proteins (Figure 7). Coordinated changes in the expression profile of genes encoding enzymes in carotenoid and chlorophyll biosynthesis were observed in *ntssu II* transgenic lines (Figure 7). Synthesizing from the common precursor GGPP, carotenoid, and chlorophyll trend to keep in a coordinated manner and in stable proportions [9]. Any disturbance of carotenoid content will be reflected in the production of photosystem components [19]. Coordinated changes of *NtSSU II* with other genes related to carotenoid and chlorophyll biosynthesis benefit efficiently regulate carotenoid and chlorophyll biosynthesis as well as photosynthetic efficiency in higher plants. Although multiple members of SSUs, GGPPS, and PSYs consisted in tobacco, the newly identified NtSSU II could especially interact with NtGGPPS1 and NtPSY1, thereby enabling the enzyme complex to guide GGPP toward the metabolic pathway of carotenoids biosynthesis. NtSSU II roles as a regulator of NtGGPPS1, which exhibited improvement of carotenoid production compared with NtGGPPS1 homodimers in *N*. *tabacum* (Figure 8).

## 4. Materials and Methods

### 4.1. Plant Materials and Growth Conditions

*N. tabacum* cultivar K326 was introduced by Northup King Seed Company (America) to Yunnan province (China) in 1985 and is preserved at Henan Agricultural University for scientific research. Both *N. tabacum* K326 and *N. benthamiana* were planted in a greenhouse of Henan Agricultural University, with a photo-cycle of 16 h light/8 h dark and the relative humidity (60 ± 2)% as growth conditions previously described [34]. The materials, including leaves, stems, roots, and flowers of *N. tabacum,* were collected and stored at −80 °C for further experiment. For high light treatment, six-weeks tobacco plants were exposed to high light (1250 μmol photons m^−2^ s^−1^). For low light treatment, 6-week tobacco plants were exposed to low light (100 μmol photons m^−2^ s^−1^), and plants exposed to normal light conditions (350 μmol photons m^−2^ s^−1^) were used as the controls. The growth conditions of transgenic tobacco cultured in Murashige–Skoog (MS) medium were 16 h/8 h of the light–dark cycle, and the temperature was (23 ± 2) °C.

### 4.2. Identification and Analysis of NtSSU

The homologous proteins of SSU in rice were identified from the TIGR rice database (http://rice.uga.edu/, accessed on 7 July 2020) and Arabidopsis TAIR (http://arabidopsis.org, accessed on 7 July 2020) database, which were used to search the homologous protein sequences of SSU in *N. tabacum* in the tobacco genome database by BLASTP (e < 0.001). The software SMART version 9 (http://smart.embl-heidelberg.de/, accessed on 15 July 2020) was used to confirm that all candidate SSU had typical conserved domains. The GSDS version 2.0 online software (http://gsds.cbi.pku.edu.cn/, accessed on 15 July 2020) was performed to visualize gene structure. The SSU protein sequences from different species were aligned by the Clustal W program. Mega 7.0 was performed for sequence alignment and inference of evolutionary tree by the neighbor-joining algorithm.

### 4.3. Expression Pattern of NtSSU in Various Tissues and in Response to Light Stresses

The materials from different plant parts were collected, including root. stem, the 3rd leaf, the 5th leaf, the 10th leaf, the 15th leaf, and flower at resettling stage, vigorous growing stage, and blooming stage, respectively. Four-week tobacco plants were selected for light treatments. The tobacco was treated with high light (1000 μmol m^−2^ s^−1^), and the leaves were collected after 1 h, 2 h, 4 h, and 6 h high light treatment. For low light treatment, the tobacco was treated by low light (100 μmol m^−2^ s^−1^), and the leaves were collected after 1 h, 2 h, 4 h, 6 h, and 8 h low light treatment. Total RNA was isolated from plant tissues using the Plant Total RNA Isolation Kit (Sangon, Shanghai, China), and the first-strand cDNA was synthesized by RNA Reverse Transcription Kit (Thermo Fisher, Waltham, MA, USA). The expression levels of genes were detected via quantitative real-time PCR (qPCR) using 26s rRNA as the reference gene. The reaction system and conditions were consulted with the manufacturer’s protocol [34]. Three biological replicates were conducted for each sample. The relative expression levels of genes were calculated using the 2^−∆∆CT^ method. The gene-specific primers used for qPCR are listed in Table S2.

### 4.4. Subcellular Localization of NtSSUs

The open reading frames (ORFs) of NtSSU gene were amplified by PCR from the cDNA of *N. tabacum* leaves with the primers containing *Spe*I and *Kpn*I restriction sites at 5-terminal (Table S1). The modified vector pCAMBIA sup1300-GFP vector was used to express NtSSU as our previous method [19]. The NtSSU II was inserted into pCAMBIA sup1300-GFP vector by *Spe*I and *Kpn*I restriction enzymes, which was expressed with a GFP-tag at the C terminus under the control of 35S RNA CaMV promoter (Table S2). The recombinant vectors were transferred into *Agrobacterium* (GV3101) by electroporation and cultured at 30 °C for 2 days. The positive *Agrobacterium* was cultured in 10 mL YEB medium and suspended with 10 mM MgCl_2_ (including 120 μM acetosyringone, AS) and adjusted OD600 to 0.6. Then, *Agrobacterium* was used to infect *N. benthamiana* plants with about five leaves for the transient expression of NtSSU II-GFP protein. Fluorescence observation was performed by a FluoView FV 1000 laser scanning confocal microscopy system (Olympus, Tokyo, Japan) after 48 h inoculation [19]. The excitation wavelength for GFP was 515 nm, with emission filters of 530–560 nm. Chlorophyll autofluorescence was monitored using a 635 nm excitation wavelength and a 650–750 nm detection window.

### 4.5. Bacterial Pigment Complementation Assay

The NtSSU II without putative transit peptides truncated were cloned into pET28a (Table S2). To determine GGPPS activity, the recombinant vectors of pET28a-NtSSU II were co-transformed with pET32b-NtGGPPSs and pAC-94N into *E. coli* BL21(DE3) cells [19]. The positive clones co-transformed were selected on Luria-Bertani (LB) medium containing ampicillin (100 μg/mL), 50 μg/mL kanamycin (50 μg/mL), and chloramphenicol (50 μg/mL), and cultured in liquid LB medium overnight. When the culture reached A600 nm of 0.3, recombinant proteins were induced with 1 mmol/L isopropy-β-d-thiogalactoside (IPTG) at 20 ℃ for 20 h. The cell pellet was centrifuged in 96-well plates for photography, and 900 μL 80% (*v/v*) acetone was added. The extraction mixture was incubated at 30 ℃ for 30 min. After centrifugation, the supernatant was analyzed for absorbance at 440 nm (A440) to measure β-carotene contents with a microplate reader (Thermo Fisher, Waltham, MA, USA) using an empty vector as control [19].

### 4.6. Biomolecular Fluorescence Complementation (BiFC) Assay

According to our previous method [19], the ORFs of a pair of genes were inserted into pCNHP-neYFP-N1 and pCNHP-ceYFP-N1, respectively, by a ClonExpress MultiS One Step Cloning Kit (Vazyme, Nanjing, China) (Table S2). For each pair, 10 μg of each recombinant plasmid were mixed and transferred into the *A. tumefaciens* GV3101 strain by electroporation. Six-week-old leaves of *N. benthamiana* were infiltrated for transient expression of these recombinant plasmids using negative *Agrobacterium* cells as control. A laser scanning confocal microscopy system (Olympus, Tokyo, Japan) was used for fluorescence observation. The excitation wavelength for EYFP was 488 nm, with emission filters of 490–550 nm. Chlorophyll autofluorescence was monitored using a 635 nm excitation wavelength and a 650–750 nm detection window.

### 4.7. Construction of Gene Editing Vectors and Identification of Homozygous ntssu II Mutants

The CRISPR/Cas9 vectors pSHE401 were kindly provided by Prof. Qijun Chen from China Agricultural University, which were successfully modified to enable the precise mutation of genes in tobacco [36,40]. The design of target sites and the construction of CRISPR/Cas9 vectors for NtSSU II were carried out according to the Mutation Sites Based Specific Primers Polymerase Chain Reaction (MSBSP-PCR) method [36]. Briefly, the exon and intron sequence of NtSSU II was studied, and target sites were identified using CRISPR Multi Targeter (http://www.multicrispr.net/index.html, accessed on 20 October 2020) based on the multiple PAM (NGG or CCN) sites. The optimal sgRNA sequence was selected close to the 5-terminal of the *NtSSU II* CDS (Table S2). The double-stranded DNA was generated by a pair of primers as sgRNA-SSU II-F and sgRNA-SSU II-R (Table S2) using our previous method [36,40]. The recombinant vectors pHSE401-SSU II were constructed and transformed individually into tobacco callus. The tobacco seeds were harvested and planted in greenhouse with a photo-cycle of 16 h light/8 h dark and the relative humidity (60 ± 2)% as growth conditions. After self-pollination, the transgenic-positive plants were identified based on the presence of the kanamycin resistance gene sequence. The T1 generation plants were obtained, and the mutation sites of the gene-editing positive plants were identified by two pairs of primers (Table S2) according to the MSBSP-PCR method [36,40]. The PCR reactions for screening positive plants were as follows: if the first round of PCR reaction cloned the target products (IsgRNA-SSU II-F and IsgRNA-SSU II-R), and the second round of PCR reaction amplified no target products (sgRNA-NtSSU II-F and IsgRNA-NtSSU II-R) (Table S2), and then the positive bacterial solution was harvested. DNA sequencing was performed to screen the same deletions that were mutated in both homologous regions on two homologous chromosomes (Tsingke, Beijing, China). Finally, *ntssu II* homozygous mutant lines were screened by the MSBSP-PCR method [36,40].

### 4.8. Extraction and Determination of Chlorophyll and Carotenoid

The chlorophyll and carotenoids were extracted and measured from transgenic tobacco as described previously [33]. Briefly, 50 mg of leaves from transgenic tobacco were ground into a fine powder and mixed with 80% (*v/v*) acetone. These mixtures were immediately shocked at 4 ℃ to bleach completely. The supernatant was harvested by centrifugation at 9000 rpm for 2 min at 4 ℃, and the absorbance values at the wavelengths of A663, A647, and A470 were measured by the microplate reader (Thermo Fisher, Waltham, MA, USA) using 80% (*v/v*) acetone as control. Chlorophyll and carotenoid contents were calculated as follows: Chlorophyll a = 12.25 × A663 − 2.79 × A647; Chlorophyll b = 21.50 × A647 − 5.10 × A663; Total chlorophyll = 7.15 × A663 + 18.71 × A647; Carotenoids = (1000 × A470 − 1.82 × Chl a − 85.02 × Chl b)/198.

### 4.9. Statistical Analysis

A completely randomized block design with three biological replicates was applied for each experiment. The data were represented by the mean ± standard deviation from three biological samples and further analyzed using the SPSS statistical package (version 8.0) for statistical analysis.

## 5. Conclusions

Different from SSU II reported in rice and pepper, three members, including NtSSU II, NtSSU I-1, and NtSSU I-2, consisted in tobacco, and only NtSSU II, especially roles as the stimulator of carotenoid biosynthesis in response to light treatments. Our data confirm the specific regulatory role of NtSSU II for the carotenoid biosynthesis in the polyploid plants’ tobacco, which contains multiple members of SSUs, GGPPS, and PSYs. The essential nature of NtSSU II was confirmed genetically based on the severe developmental phenotype and expression analysis of the *ntssu II* null mutants. Furthermore, the formation of multi-enzyme complexes containing NtGGPPS1, NtSSU II and particular GGPP-consuming enzymes NtPSY1 enzymes could be an additional mechanism besides the gene co-expression to control metabolic flux to specific carotenoids biosynthetic pathways. Exploring the regulation of GGPPS activity by NtSSU II has great theoretical significance in improving carotenoid content, providing a solid foundation for enhancing photosynthesis efficiency in polyploid plants.

## Data Availability

Not applicable.

## Supplementary Materials

The following supporting information can be downloaded at: https://www.mdpi.com/article/10.3390/ijms24020992/s1.
